# Editorial: Advances in methods and tools for multi-omics data analysis

**DOI:** 10.3389/fmolb.2023.1186822

**Published:** 2023-04-24

**Authors:** Ornella Cominetti, Sumeet Agarwal, Sergio Oller-Moreno

**Affiliations:** ^1^ Nestlé Research Center, Lausanne, Switzerland; ^2^ Indian Institute of Technology Delhi, New Delhi, India; ^3^ Institute for Bioengineering of Catalonia (IBEC), The Barcelona Institute of Science and Technology, Barcelona, Spain

**Keywords:** multi-omics, personalized medicine, machine learning, integrative omics, deep generative models, protein protein interaction (PPI) network, EATRIS European infrastructure for translational medicine

Multi-omics data analysis is a rapidly growing field with significant potential in personalized medicine. Despite the many advances in this domain, there are still important challenges that need to be addressed, such as standardization of methods and limitations in interpreting results.

The Research Topic “*Advances in Methods and Tools for Multi-Omics Data Analysis”* showcases novel techniques and tools, including machine/deep learning tools, multi-factor analysis, Bayesian statistics, network-based models, and computational approaches for network-based integrative multi-omics analysis.

This article Research Topic comprises one perspective article, which addresses the translational challenges of multi-omics research in the realm of European personalized medicine, four review articles covering challenges in the path towards achieving precision medicine in cancer treatment and immuno-oncology, computational approaches for network-based integrative multi-omics analysis, deep generative models for learning joint embeddings of single-cell multi-omics data, and methods for characterization and visualization of protein–protein interaction networks in a multi-omics integration context.

Additionally, there are five original research articles ranging from the presentation of a new statistical framework for the analysis of longitudinal multi-omics data to the application of multi-omics methods to different diseases such as cancer, cutaneous melanoma, inflammatory diseases, myocardial dysfunction, and tuberous sclerosis complex-related angiomyolipoma.

Various machine learning (ML) techniques that can be used to integrate multi-omics data are discussed in this Research Topic. Network-based diffusion/propagation methods and multiview/multi-modal ML are two such techniques that can exploit information captured in each omics dataset and infer associations between different data types. Deep learning methods are an example of multiview/multi-modal learning, which can capture complex non-linear associations in a multi-layered manner.

Complex longitudinal omics datasets require new statistical approaches for their analysis, and ALASCA, a new package presented by Jarmund, Madssen and Giskeødegård, shows a promising framework for tackling the longitudinal and multivariate nature of multi-omics studies, as well as covariate adjustment. In contrast, Magnusson et al. used a different approach for longitudinal omics data analysis: they applied linear mathematical mixed time-delayed splice variant models to predict protein abundances from mRNA expression.

The review article by Brombacher and colleagues provides a systematic overview of current deep generative models (DGM)-based approaches for learning joint embeddings from multi-omics data and illustrates how small sample sizes impact the amount of information that can be recovered from such datasets. Specifically, the review examines how the performance of popular DGM-based approaches to infer joint low-dimensional representations is influenced by varying numbers of cells, which is particularly relevant at the stage of designing an experiment.

Robin and colleagues discuss the role of protein-protein interactions (PPIs) in cellular mechanisms and the construction of PPI networks. PPIs are involved in physical and biochemical processes in structured environments and can be constructed using prediction methods and high-throughput experiments. Computational methods have emerged as a promising way to identify PPIs, and integration methods can be used to filter false interactions. Visualization is a key step in analyzing PPI networks, but the complexity of proteomes in different organisms presents a challenge.

The article by Oldoni and colleagues summarizes the outcome of the 2021 European Infrastructure for Translational Medicine (EATRIS)-Plus Multi-omics Stakeholder Group workshop. This multidisciplinary and cross-institutional working group aims to become a European reference group for implementing personalized medicine across Europe. This perspective article discusses the potential of precision medicine in healthcare and the challenges associated with it, such as moving beyond genomics to integrated multi-omics and multi-modal complex biomarker generation, new technologies and digital health, data standardization to enable multi-modal integration and AI-supported drug modeling, variability in omics data at source, data privacy and regulatory aspects, and economic implications.

Moreover, the Research Topic includes original research articles that demonstrate the application of multi-omics in various diseases. For example, Wang and colleagues present a study where ultra-performance liquid chromatography-mass spectrometer (UPLC-MS) was used to measure plasma proteins and metabolites in patients with renal cysts, sporadic angiomyolipoma, and tuberous sclerosis complex (TSC)-related angiomyolipoma before and after immunosuppressant treatment, with the aim of finding potential diagnostic and prognostic biomarkers as well as revealing the underlying mechanism of TSC tumorigenesis.

In another contribution, Wang et al. applied Least Absolute Shrinkage and Selection Operator (LASSO) and Support Vector Machine-Recursive Feature Elimination (SVM-RFE) algorithms to identify a cancer cell stemness feature, identifying 3 specific subtypes of melanoma with different survival outcomes.

One of the key challenges in multi-omics data analysis is the combination of different data types to identify composite biomarker signatures. This merging is complicated by the fact that multi-omics data often needs to be coupled with other data, such as imaging data, phenotypic data, and medical data (Electronic Health Records and patient-related outcomes). The integration of multi-omics and multi-modal data marks a significant step closer to personalized medicine, although many challenges remain before these biomarkers can be fully implemented in routine clinical care. The contributions in this Research Topic represent a small but solid base of step towards achieving these goals.

